# Outcomes of androgenetic alopecia treated with dutasteride mesotherapy: A case series

**DOI:** 10.1016/j.jdcr.2024.09.024

**Published:** 2024-10-15

**Authors:** Ambika Nohria, Deesha Desai, Maria Salomé Páez-García, Kristen I. Lo Sicco, Jerry Shapiro

**Affiliations:** a Ronald O. Perelman Department of Dermatology, New York University Grossman School of Medicine, New York, New York; b University of Pittsburgh School of Medicine, Pittsburgh, Pennsylvania; c Fundación Universitaria de Ciencias de la Salud, Bogotá, Colombia

**Keywords:** alopecia, androgenetic alopecia, dutasteride, mesotherapy

## Introduction

The pathogenesis of androgenetic alopecia (AGA) involves the binding of dihydrotestosterone to androgen receptors in hair follicles, causing follicular miniaturization and a shortened growth phase.^1^

AGA treatments may aim to reduce serum and scalp dihydrotestosterone levels to inhibit this process. These medications include 5-αa-reductase inhibitors finasteride, which is currently US Food and Drug Administration–approved for the treatment of AGA in males, and dutasteride, an off-label treatment for AGA.^2^ Oral dutasteride, originally approved for benign prostatic hyperplasia, may be an efficacious AGA treatment modality; however, systemic use can lead to side effects including sexual dysfunction and gynecomastia.^2^^,^^3^

Mesotherapy, injection of active substances directly into the scalp, has emerged as an alternative approach to administering dutasteride while limiting side effects, although this method of drug delivery is not currently approved by the US Food and Drug Administration. Despite its widespread international use, dutasteride mesotherapy remains limited in the United States.^4^, ^5^, ^6^, ^7^, ^8^ This study aims to evaluate its efficacy in 10 patients.

## Methods

This study is an NYU Langone Health Institutional Review Board–approved (i23-00157; approved March 14, 2023) retrospective analysis of 10 patients seen at NYU Langone Health dermatology. Dutasteride sterile intradermal injectable was compounded at a strength of 0.1% as a water-containing proprietary solution without propylene glycol, ethanol, dimethylsulfoxide and oil. Injections were performed with a 0.5-inch 30-gauge needle, with injections spaced 0.5 to 1 cm apart. A total of 1.25 mL of mesotherapy was injected (0.05 mL per injection) approximately every 3 months. Hair density and width were measured at the frontal scalp (12 cm from the glabella) and vertex (20-24 cm from the glabella) using the Canfield HairMetrix device. Patient charts were reviewed in Epic and relevant information was obtained from provider notes. Changes in hair density and width were compared using a *t* test or Mann–Whitney test.

## Results

Ten patients, four men and six women, received dutasteride mesotherapy. The mean age at the start of mesotherapy was 50.6 years (range, 22-80 years). All patients were receiving various concomitant topical and oral medications for AGA treatment started at least 4 months prior to mesotherapy (Table I). Patients were evaluated after each round of mesotherapy and received a total of either 1, 2, or 3 rounds.

**Table I:** Patient demographics and hair counts

Case	Sex	Age atdutasteride start (y)	Concomitant medications	Baseline hair counts before starting dutasteride	Hair counts after first round dutasteride	Hair counts after second round dutasteride	Hair counts after third round dutasteride	Overall delta hair counts
Case 1	M	52	Oral finasteride 1 mg/d, oral minoxidil 2.5 mg/d, topical minoxidil 5% solution twice a day	@22 cm from glabella: 96 hairs/cm^2^, 43.5 μm	@22 cm from glabella: 132 hairs/cm^2^, 51.4 μm	@22 cm from glabella: 119 hairs/cm^2^, 49.2 μm	-	@22 cm from glabella: +23 hairs/cm^2^, +5.7 μm
Case 2	M	35	Oral finasteride 1 mg/d, oral minoxidil 7.5 mg/d, topical minoxidil 5% solution twice a day, topical Polaris research NR11 daily, PRP every 3 mo	@12 cm from glabella: 159 hairs/cm^2^, 55.7 μm @24 cm from glabella: 168 hairs/cm^2^, 50.9 μm	@12 cm from glabella: 181 hairs/cm^2^, 55.8 μm @24 cm from glabella: 203 hairs/cm^2^, 48.6 μm	@12 cm from glabella: 159 hairs/cm^2^, 51.0 μm @24 cm from glabella:162 hairs/cm^2^, 49.4 μm	-	@12 cm from glabella: 0 hairs/cm^2^, −4.7 μm @24 cm from glabella: −6 hairs/cm^2^, −1.5 μm
Case 3	F	56	Iron supplementation, oral minoxidil 2.5 mg/d, PRP every 12 mo	@12 cm from glabella: 227 hairs/cm^2^, 62 μm	@12 cm from glabella: 230 hairs/cm^2^, 62 μm	@12 cm from glabella: 242 hairs/cm^2^, 62 μm	-	@12 cm from glabella: +15 hairs/cm^2^, 0 μm
Case 4	F	22	Oral minoxidil 5mg/d, oral spironolactone 100 mg twice a day, topical minoxidil 5% solution twice a day	@12 cm from glabella: 194 hairs/cm^2^, 54.1 μm @20 cm from glabella: 195 hairs/cm^2^, 62.3 μm	@12 cm from glabella: 188 hairs/cm^2^, 57.4 μm @20 cm from glabella: 192 hairs/cm^2^, 55.5 μm	@12 cm from glabella: 207 hairs/cm^2^, 56.1 μm @20 cm from glabella: 194 hairs/cm^2^, 60.0 μm	-	@12 cm from glabella: +13 hairs/cm^2^, +2 μm @20 cm from glabella: −1 hairs/cm^2^, −2.3 μm
Case 5	F	34	Oral minoxidil 5 mg/d, oral finasteride 2.5 mg/d5, iron supplementation, bicalutamide 50 mg/d switched to spironolactone 100 mg/d after second round of mesotherapy	@12 cm from glabella: 166 hairs/cm^2^, 65.0 μm @24 cm from glabella: 162 hairs/cm^2^, 68.2 μm	@12 cm from glabella: 198 hairs/cm^2^, 63.2 μm @24 cm from glabella: 152 hairs/cm^2^, 67.0 μm	@12 cm from glabella: 193 hairs/cm^2^, 62.4 μm @24 cm from glabella: 164 hairs/cm^2^, 62.5 μm	@12 cm from glabella: 166 hairs/cm^2^, 60.9 μm @24 cm from glabella: 170 hairs/cm^2^, 63.9 μm	@12 cm from glabella: 0 hairs/cm^2^, −4.1 μm @24 cm from glabella: +8 hairs/cm^2^, −4.3 μm
Case 6	M	47	Oral minoxidil 5 mg/d, oral finasteride 5 mg/d, compounded solution of topical minoxidil 7% and finasteride 0.25% twice a day	@12 cm from glabella: 143 hairs/cm^2^, 74.4 μm @24 cm from glabella: 199 hairs/cm^2^, 43.5 μm	@12 cm from glabella: 153 hairs/cm^2^, 73.7 μm @24 cm from glabella: 162 hairs/cm^2^, 50.8 μm	@12 cm from glabella: 139 hairs/cm^2^, 75.8 μm @24 cm from glabella: 151 hairs/cm^2^, 77.7 μm	-	@12 cm from glabella: −4 hairs/cm^2^, +1.4 μm @24 cm from glabella: −48 hairs/cm^2^, +34.2 μm
Case 7	F	64	Oral finasteride 5 mg/d, oral minoxidil 2.5 mg/d, topical minoxidil 5% solution twice a day	@12 cm from glabella: 164 hairs/cm^2^, 76.6 μm	@12 cm from glabella: 159 hairs/cm^2^, 68.0 μm	@12 cm from glabella: 180 hairs/cm^2^, 69.7 μm	@12 cm from glabella: 162 hairs/cm^2^, 69.5 μm	@12 cm from glabella: −2 hairs/cm^2^, −7.1 μm
Case 8	F	58	Oral minoxidil 1.25 mg/d, oral finasteride 5 mg/d, topical minoxidil 5% solution twice a day, PRP every 3 mo	@12 cm from glabella: 168 hairs/cm^2^, 71.8 μm	-	@12 cm from glabella: 153 hairs/cm^2^, 75.5 μm	-	@12 cm from glabella: −15 hairs/cm^2^, +3.7 μm
Case 9	F	80	Oral finasteride 2.5 mg/d, oral minoxidil 2.5 mg/d, topical minoxidil 5% solution twice a day, PRP every 2-3 mo	@12 cm from glabella: 236 hairs/cm^2^, 44.0 μm @24 cm from glabella: 211 hairs/cm^2^, 46.2 μm	@12 cm from glabella: 177 hairs/cm^2^, 57.8 μm @24 cm from glabella: 208 hairs/cm^2^, 55.6 μm	-	-	@12 cm from glabella: −59 hairs/cm^2^, +13.8 μm @24 cm from glabella: −3 hairs/cm^2^, +9.4 μm
Case 10	M	58	Oral finasteride 1 mg/d, oral minoxidil 5 mg/d, compounded solution of topical minoxidil 8% and finasteride 0.25% twice a day	@12 cm from glabella: 127 hairs/cm^2^, 60.7 μm @24 cm from glabella: 99 hairs/cm^2^, 66.4 μm	@12 cm from glabella: 120 hairs/cm^2^, 55.2 μm @24 cm from glabella: 110 hairs/cm^2^, 63.4 μm	-	-	@12 cm from glabella: −7 hairs/cm^2^, −5.5 μm @24 cm from glabella: +11 hairs/cm^2^, −3 μm

*F*, Female; *M*, male; *PRP*, platelet rich plasma scalp injections.

Two patients demonstrated improvement in hair density, 4 patients had a lack of response, and 4 patients had variation in response between the frontal scalp and vertex.

**Fig 1:**
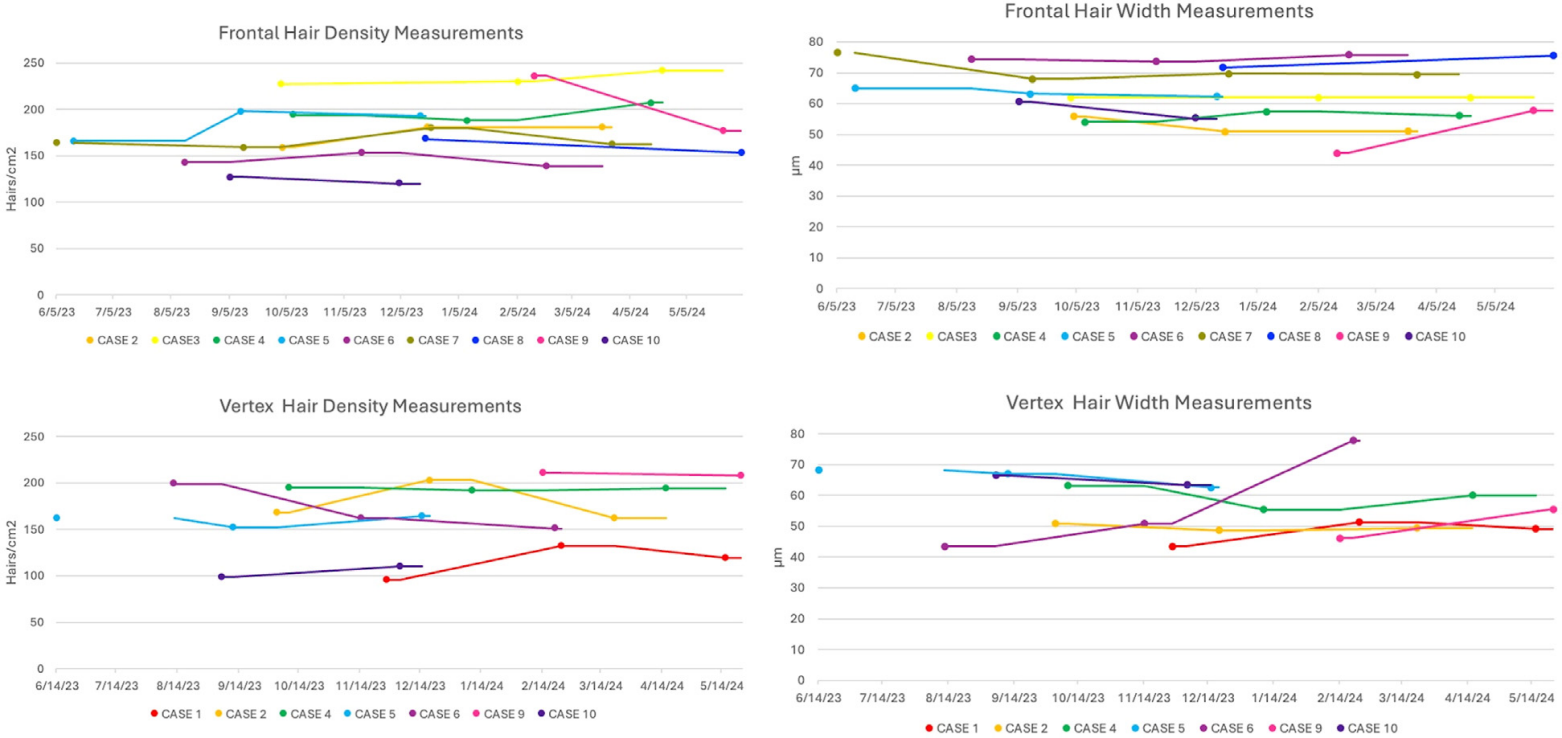
Frontal and vertex hair density and width across treatment sessions. Dots represent treatment visits. Lines represent moving means between measurements at each visit. Frontal measurements were taken at 12 cm from the glabella. Vertex hair measurements were taken at 20 to 24 cm from the glabella.

Three patients demonstrated improvement in hair width, 5 patients had a lack of response, and 2 patients had variation in response between the frontal scalp and vertex (Table I, Fig 1).

Overall, the mean time between baseline and final evaluation was 176.3 days. For our overall cohort, at the frontal scalp, patients demonstrated a mean loss of 6.5 hairs/cm^2^ and 0.06 μm. At the vertex, there was a mean loss of 2.3 hairs/cm^2^ but a gain of 5.5 μm. There was no significant change in hair count or diameter at the frontal scalp (*P* = .67; *P* = .72) or at the vertex (*P* = .96; *P* = .37; Table II).

**Table II:** Cohort and subcohort treatment outcomes

Cohort	Change in frontal hair density (hairs/cm^2^)	Change in vertex hair density (hairs/cm^2^)	Change in frontal hair width (μm)	Change in vertex hair width
Overall cohort (*N* = 10)	−6.5 (20.5)	−2.3 (20.8)	−0.06 (6.1)	5.5 (12.6)
	*P* = .67	*P* = .72	*P* = .96	*P* = .37
Men (*n* = 4)	−3.7 (3.5)	−5 (31.0)	−2.9 (3.8)	8.9 (17.3)
Women (n = 6)	−8 (27.3)	1.3 (5.9)	1.4 (7.3)	0.9 (7.4)
Age <50 y (*n* = 4)	2.3 (7.4)	−11.8 (24.6)	−1.4 (3.5)	6.5 (18.5)
Age >50 y (*n* = 6)	−13.6 (27.7)	10.3 (13.0)	0.98 (8.4)	4.0 (6.4)
1 round of mesotherapy (*n* = 2)	−33 (36.8)	4 (9.9)	4.2 (13.6)	3.2 (8.8)
2 rounds of mesotherapy (*n* = 6)	1.8 (12.4)	−8 (29.5)	0.48 (3.2)	9.0 (17.2)
3 rounds mesotherapy of (*n* = 2)	−1 (1.4)	8	−5.6 (2.1)	−4.3

Values listed are means with SDs. *P* values were determined using paired *t* test.

At the frontal scalp for women there was a mean loss of 8 hairs/cm^2^ and gain of 1.4 μm, and for men a mean loss of 3.7 hairs/cm^2^ and loss of 5 μm. At the vertex, for women there was a mean gain of 1.3 hairs/cm and gain of 0.9 μm, and for men a mean loss of 5 hairs/cm and gain of 8.9 μm.

At the frontal scalp, there was a mean loss of 13.6 hairs/cm^2^ and gain of 0.98 μm in patients aged >50 years and a mean gain of 2.3 hairs/cm^2^ and loss of 1.4 μm in patients aged <50 years. At the vertex, there was a mean loss of 10.3 hairs/cm^2^ and gain of 4 μm in patients aged >50 years and a mean loss of 11.8 hairs/cm^2^ and gain of 6.5 μm in patients aged<50 years.

We compared outcomes between those who received 1, 2, or 3 rounds of mesotherapy. At the frontal scalp, for those who received 1 round of injections, there was a mean loss of 33 hairs/cm^2^ and gain of 4.2 μm, for 2 rounds a mean gain of 1.8 hairs/cm^2^ and gain of 0.48 μm, and for 3 rounds, a mean loss of 1 hairs/cm^2^ and loss of 5.6 μm. At the vertex, for those who received 1 round of injections there was a mean gain of 4 hairs/cm^2^ and gain of 3.2 μm, for 2 rounds a mean loss of 8 hairs/cm^2^ and gain of 9 μm; and for 3 rounds, a mean gain of 8 hairs/cm^2^ and loss of 4.3 μm. Direct statistical comparison between genders, age groups, and rounds of mesotherapy received was not possible due to the small sample size (Table II).

No adverse effects from mesotherapy were reported by any of the 10 patients.

## Discussion

This case series evaluates the outcomes of dutasteride mesotherapy in 10 patients. The findings suggest that dutasteride mesotherapy may not produce significant improvements in hair density and diameter. There were no statistically significant changes in any measurements between visits with highly variable treatment responses. Some patients experienced positive results at the frontal scalp, but there was an overall loss of density and width at the vertex, emphasizing inconsistency in treatment response.

The literature on dutasteride mesotherapy remains limited, with varied documented outcomes. In a similar study of 6 men and women receiving 1 mL of 0.01% dutasteride mesotherapy, all patients demonstrated improvement in AGA with increased hair density and diameter measurements.^5^ Other studies employing 0.05% dutasteride in men and women reported improvement in 30% to 93% of patients, highlighting the potential inconsistent outcomes of this treatment modality.^6^^,^^7^ A recent meta-analysis comparing intralesional with oral dutasteride reported mean improvement in hair counts to be greater with oral therapy; however, statistical comparison was not possible given the lack of reported outcomes in mesotherapy trials.^8^

There are numerous possible explanations for the limited success seen in this case series. First, the treatment duration and frequency may not have been sufficient to elicit enough of a response. In prior studies that demonstrated positive outcomes, the patients received anywhere from 7 to 12 rounds of injections, initially spaced just 1 week apart.^5^, ^6^, ^7^ In our series, 2 patients received 3 rounds of mesotherapy, 6 patients received 2 rounds, and 2 patients received just 1 round, all spaced approximately 3 months apart. When comparing between these 3 groups, it did not appear that receiving more rounds of mesotherapy improved outcomes; however, our sample size limited our ability to make direct statistical comparisons. Nevertheless, this variation in frequency and timing of mesotherapy could be a possible contributor to our observed lack of response. Additionally, the formulation of the dutasteride solution must also be considered when discussing the varied outcomes of these patients. Prior rare cases of paradoxical nonscarring alopecia after dutasteride mesotherapy have been reported due to an acute reaction to ethanol solvent.^9^ This, however, is unlikely to have contributed to poor response among our patients as the solvent used in our formulation contains no ethanol, oil or propylene glycol. Lastly, dutasteride mesotherapy remains a new treatment modality for AGA, with mixed results reported in literature. The lack of definitive improvement shown in this series may be reflective of a lack of efficacy for treatment of this condition; however, additional studies are necessary.

Importantly, none of our patients reported adverse effects from dutasteride mesotherapy. This aligns with prior studies where most side effects were mild and self-limited, primarily involving pain during the procedure.^6^, ^7^, ^8^ This highlights the potential advantage of mesotherapy over oral administration in minimizing exposure to systemic side effects.

Although these results were not positive overall, there are several key limitations that must be considered. The cohort included was small, the duration of treatment and follow-up was relatively short, and severity of alopecia was not controlled for. Additionally, not every patient received more than 1 treatment of mesotherapy, and perhaps may have demonstrated improvement with further rounds of treatment. Despite these limitations, our results highlight that dutasteride mesotherapy warrants further prospective research before concluding that it is a consistently beneficial treatment modality for AGA and should be reserved as a nonfirst line therapy.

## Conflicts of interest

Dr Lo Sicco has been an investigator for Regen Lab and is an investigator for Pfizer. Dr Lo Sicco is a consultant for Pfizer and Aquis. Dr Shapiro is an investigator and consultant for Pfizer and is a consultant for Lilly. Authors Nohria and Desai and Dr Páez-García have no conflicts of interest to declare.
